# Supplementary material for: A fault-intrusion-tolerant system and deadline-aware algorithm for scheduling scientific workflow in the cloud
Source: PeerJ Comput Sci. 2021 Nov 2;7:e747. doi: 10.7717/peerj-cs.747 (PMC8576554; doi:10.7717/peerj-cs.747)
Supplement: Supplemental Information 6 [file peerj-cs-07-747-s006.zip › Myproject1.1/docs/org/cloudbus/cloudsim/util/ExecutionTimeMeasurer.html]

ExecutionTimeMeasurer


JavaScript is disabled on your browser.


- Overview
- Package
- Class
- Use
- Tree
- Deprecated
- Index
- Help

- Prev Class
- Next Class

- Frames
- No Frames

- All Classes

- Summary:
- Nested |
- Field |
- Constr |
- Method

- Detail:
- Field |
- Constr |
- Method


org.cloudbus.cloudsim.util

## Class ExecutionTimeMeasurer

- java.lang.Object
- - org.cloudbus.cloudsim.util.ExecutionTimeMeasurer

- ---

    

  ```
  public class ExecutionTimeMeasurer
  extends java.lang.Object
  ```

  The class for measuring the execution time.

  Since:
  :   CloudSim Toolkit 3.0

- - ### Constructor Summary

    Constructors

    Constructor and Description

    `ExecutionTimeMeasurer()`
  - ### Method Summary

    Methods

    | Modifier and Type | Method and Description |
    | --- | --- |
    | `static double` | `end(java.lang.String name)` End. |
    | `static java.util.Map<java.lang.String,java.lang.Long>` | `getExecutionTimes()` Gets the execution times. |
    | `static void` | `start(java.lang.String name)` Start. |

    - ### Methods inherited from class java.lang.Object

      `clone, equals, finalize, getClass, hashCode, notify, notifyAll, toString, wait, wait, wait`

- - ### Constructor Detail


    - #### ExecutionTimeMeasurer

      ```
      public ExecutionTimeMeasurer()
      ```
  - ### Method Detail


    - #### start

      ```
      public static void start(java.lang.String name)
      ```

      Start.

      Parameters:
      :   `name` - the name


    - #### end

      ```
      public static double end(java.lang.String name)
      ```

      End.

      Parameters:
      :   `name` - the name

      Returns:
      :   the double


    - #### getExecutionTimes

      ```
      public static java.util.Map<java.lang.String,java.lang.Long> getExecutionTimes()
      ```

      Gets the execution times.

      Returns:
      :   the execution times


- Overview
- Package
- Class
- Use
- Tree
- Deprecated
- Index
- Help

- Prev Class
- Next Class

- Frames
- No Frames

- All Classes

- Summary:
- Nested |
- Field |
- Constr |
- Method

- Detail:
- Field |
- Constr |
- Method
